# Structure and scaling of the middle ear in domestic dog breeds

**DOI:** 10.1111/joa.14049

**Published:** 2024-04-11

**Authors:** Matthew J. Mason, Madaleine A. Lewis

**Affiliations:** ^1^ Department of Physiology, Development & Neuroscience University of Cambridge Cambridge UK

**Keywords:** middle ear, ossicles, dog, hearing, allometry

## Abstract

Although domestic dogs vary considerably in both body size and skull morphology, behavioural audiograms have previously been found to be similar in breeds as distinct as a Chihuahua and a St Bernard. In this study, we created micro‐CT reconstructions of the middle ears and bony labyrinths from the skulls of 17 dog breeds, including both Chihuahua and St Bernard, plus a mongrel and a wolf. From these reconstructions, we measured middle ear cavity and ossicular volumes, eardrum and stapes footplate areas and bony labyrinth volumes. All of these ear structures scaled with skull size with negative allometry and generally correlated better with condylobasal length than with maximum or interaural skull widths. Larger dogs have larger ear structures in absolute terms: the volume of the St Bernard's middle ear cavity was 14 times that of the Chihuahua. The middle and inner ears are otherwise very similar in morphology, the ossicular structure being particularly well‐conserved across breeds. The expectation that larger ear structures in larger dogs would translate into hearing ranges shifted towards lower frequencies is not consistent with the existing audiogram data. Assuming that the audiograms accurately reflect the hearing of the breeds in question, oversimplifications in existing models of middle ear function or limitations imposed by other parts of the auditory system may be responsible for this paradox.

## Graphical abstract

Middle ear structural dimensions in dog breeds are more closely correlated with skull length than width. Although they scale with negative allometry, the St Bernard's middle ear cavity is still 14 times larger than that of a Chihuahua. Surprisingly, these substantial size differences are not reflected in existing canine audiograms.

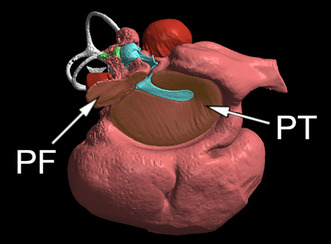

## 1 INTRODUCTION

It is well‐known that dogs respond to sound frequencies above the human hearing range, as emitted by dog‐whistles. The earliest such device was the Galton whistle, invented by Charles Darwin's half‐cousin, Francis Galton. Galton wrote, ‘Small dogs also hear very shrill notes, but large ones do not. I have walked through the streets of a town with an instrument like that which I used in the Zoological Gardens, and made nearly all the little dogs turn round, but not the large ones. At Berne, where there appear to be more large dogs lying idly about the streets than in any other town in Europe, I have tried the whistle for hours together, on a great many large dogs, but could not find one that heard it’ (Galton, 1883).

Among terrestrial mammals in general, body size and in particular interaural width is inversely related to high‐frequency hearing limits measured behaviourally (Heffner et al., 2001; Heffner & Heffner, 2003). This has been put down to the need for smaller mammals to hear higher frequencies in order to extract usable interaural intensity difference and pinna cues for sound localization. Smaller mammals also tend to produce higher‐frequency vocalizations, which may relate in part to the size of the vocal organs (Fletcher, 2004; Martin et al., 2017), so one would expect higher‐frequency hearing to be helpful in subserving intraspecific communication in smaller mammals. These arguments are consistent with the fact that smaller mammals generally have smaller middle ear structures which, based on principles of acoustics, are expected to facilitate the transmission of higher‐frequency sound to the cochlea (Hemilä et al., 1995; Mason, 2016).

These broad relationships are observed across terrestrial mammals as a whole, ranging from mice to elephants. It is more difficult to relate the relatively modest differences in behavioural audiograms to differences in ear structure in foxes, dogs and cats, which occupy a much narrower size range (Malkemper et al., 2020). Although all members of the order Carnivora, foxes, dogs and cats last shared a common ancestor nearly 60 million years ago (Eizirik et al., 2010). In an interspecific comparison such as this, evolutionary divergence in multiple aspects of the auditory system will inevitably complicate any attempt to relate differences in particular ear structures to overall function.

The ancestors of domestic dogs (*Canis lupus familiaris*) are thought to have diverged from grey wolves just 33,000 years ago, in southern East Asia (Wang et al., 2016). Efforts towards specialized breed development accelerated in the 19th century (Parker et al., 2017; Schoenebeck & Ostrander, 2013). As a result of domestication and human selection, dogs are said to show the greatest variation in body size of any terrestrial mammal (Boyko et al., 2010; Sutter et al., 2007), ranging from the Chihuahua, weighing as little as 1 kg, to the English mastiff which can reach 100 kg or more (Wayne & vonHoldt, 2012). The skull shape is also unusually variable in dogs, encompassing brachycephalic (short‐headed, e.g. Pug) through mesaticephalic to dolichocephalic (long‐headed, e.g. Saluki), high ‘foreheads’ versus low and broad muzzles versus tapering ones (Drake & Klingenberg, 2010). Dog breeds vary considerably in the shape of their cribriform plate, through which pass the olfactory nerves (Jacquemetton et al., 2021), suggesting that different skull morphologies may have an impact on sensory systems. For most traits studied, a small number of genetic variations of large effect account for much of this phenotypic variance (Boyko et al., 2010). For example, a particular *IGF1* gene allele is associated with small body size in dogs (Sutter et al., 2007), while a missense mutation in *BMP3* may underlie extreme brachycephaly, although several other genes are also involved (Schoenebeck et al., 2012). The combination of considerable size variability with significant genetic similarity within one species provides a unique opportunity to explore the link between the bony morphology of the ear and hearing while minimising the influence of many of the confounding variables which complicate interspecific studies.

**FIGURE 1:**
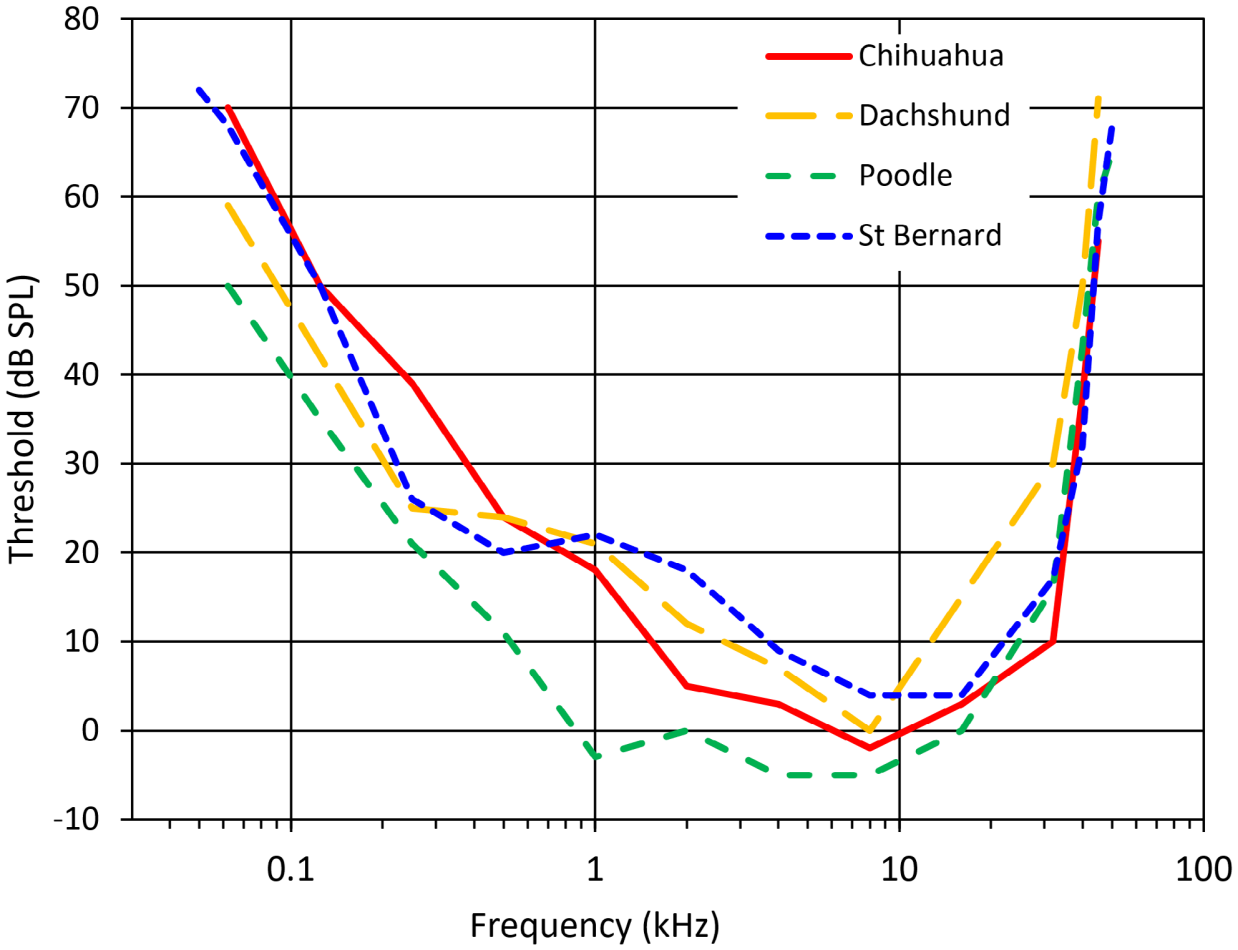
Behavioural audiograms of four dogs of different breeds, replotted from Heffner (1983).

The relationship between body size and hearing found among mammals in general is consistent with Galton's observations that smaller dogs can hear higher frequencies than larger ones. It is therefore surprising that, in a much more rigorous study of canine hearing, Heffner (1983) found no very obvious differences in the audiograms of five breeds of dogs tested behaviourally, even though these breeds spanned an order of magnitude in size from a 4.3 kg Chihuahua to a 45.5 kg St Bernard (Figure 1). One hypothesis to explain this would be that ear structures either do not scale with body size among dogs, or scale with such pronounced negative allometry that there is no measurable effect on their hearing. Eye radius in dogs, for example, was found to vary only between 9.6 and 11.6 mm, in 82 eyes from animals with skull lengths ranging from 93 to 250 mm (McGreevy et al., 2004).

As part of his canine hearing study, Heffner (1983) compared tympanic membrane areas in 15 dogs including Chihuahua and St Bernard, and found a positive correlation with body mass. Kirikae (1960) found a correlation between overall middle ear cavity volume and body mass in six dogs of between 5 and 18 kg, as did Defalque et al. (2005), who studied 18 mesaticephalic dogs between around 2 and 55 kg. In contrast, Kirikae (1960) noted that the sizes of the mesotympanic cavity, tympanic membrane and ossicular chain were ‘constant in spite of great variations in the size of the head’, in his six dogs. There is, then, disagreement in the existing literature about if and how dog middle ear components scale with body size. Since breeds were not specified in most of these studies, how the ear morphology compares in the specific breeds tested by Heffner (1983) remains unknown.

The inner ear of dogs has been looked at in more detail than the middle ear. Schweizer et al. (2017) performed a geometric morphometric comparison of the bony labyrinth of wolves, dingoes and domesticated dogs, as reconstructed from CT scans. Although the overall shape of the bony labyrinth was similar, domestic dogs showed greater variation than wolves in both bony labyrinth size and number of cochlear turns. Using the same data set, some of the variation in semicircular canal morphology between different dog breeds was subsequently found to relate to skull length (Smith & Laitman, 2021). Janssens et al. (2019) performed a separate geometric morphometric study, in which they were able to identify subtle differences in shape between dog and wolf labyrinths.

The goal of the present study was not to describe in detail the shape variations of canine ear structures, but rather to look at how the absolute dimensions of those structures believed to have an impact on hearing scale with skull size. Using micro‐computed tomography, we examined the bony structures of the ear in 17 breeds of dogs, including all five of those tested behaviourally by Heffner (1983). We also examined a mongrel and a wolf. Because dog skulls vary considerably in morphology, we looked at whether the dimensions of the examined structures correlate more closely with skull length, often used as an index of animal size in comparative studies, or with skull width, which is more obviously linked to sound localization. We then used the anatomical data obtained to make predictions about hearing based on existing models of middle ear function, and compared these predictions to the audiograms obtained by Heffner (1983). Given the comprehensive analyses mentioned above, the bony labyrinth was not examined in detail in the present study but its overall volume was considered in the scaling analysis.

## 2 MATERIALS AND METHODS

### 2.1 Specimens

Ten dog skulls were taken from the teaching collection of the Veterinary Anatomy Museum at the University of Cambridge. The breeds of eight of these dogs were indicated on the specimens; the Pekingese and Boxer skulls were identified with reference to others in the collection and photographs on the internet. A further six skulls, all labelled with the breed, were taken from the teaching collection of the Anatomic Pathology Laboratory at the Queen's Veterinary School Hospital, also at the University of Cambridge. Of these 16 specimens, the St Bernard, Poodle, Pointer and Dachshund skulls were chosen because the hearing of representatives of these breeds had been tested by Heffner (1983). The criteria for choosing the other skulls were that they should have intact bullae containing ossicles, and they should as far as possible evenly cover the widest range of skull lengths. One further skull from the Veterinary Anatomy Museum, labelled only as that of a wolf but with no species or subspecies given, was used for comparison. No information was available regarding the provenance of any of these skulls.

The frozen but otherwise intact head of a Chihuahua, representing the last of the five breeds examined behaviourally by Heffner (1983), was obtained from the Anatomic Pathology Laboratory. The 12‐year‐old female weighing 2.64 kg had been euthanized following a diagnosis of myxomatous mitral valve disease, and its body had been donated for teaching and research purposes.

The final canine specimen was obtained from the Veterinary Anatomy Dissection Laboratory at the University of Cambridge. This was the preserved half‐head of a dog presumed to be a mongrel but resembling a beagle in size and shape. It had previously been used in an undergraduate dissection class but the ears were intact. The specimen had originally come from the Carolina biological supply company and had likely been formalin‐fixed before preservation in their proprietary preservative, the primary component of which is propylene glycol. In Cambridge, the dog had been reinjected with ‘Cantabrian’ embalming fluid, based on ethanol, formaldehyde, polyethylene glycol and citricidal, to prevent mould growth.

### 2.2 CT scanning and subsequent processing

The ear regions of all 18 dog specimens plus the wolf were scanned at the Cambridge Biotomography Centre, using a Nikon XT H 225 micro‐CT scanner. For every scan, settings were optimised to account for differences in skull size and density; a scanner upgrade mid‐project also necessitated changes to the scan parameters (see Supplementary Material). Processing involved CT Agent XT 3.1.9 and CT Pro 3D XT 3.1.9 (Nikon Metrology, 2004–2013): the windowing was chosen so as to maximise visible contrast. 16‐bit tiff stacks were created, with cubic voxel side lengths of 13.6–46.3 μm. Each tiff stack was then converted to high‐quality jpeg format. The CT data that support the findings of this study are openly available in Zenodo at http://doi.org/10.5281/zenodo.10410546.

Reconstructions were produced in Stradview 7.13 (https://mi.eng.cam.ac.uk/Main/StradView), written by Graham Treece with contributions from Andrew Gee and Richard Prager, all of the Department of Engineering, University of Cambridge (Treece et al., 1999). One ear was reconstructed per specimen. In some cases, an ossicle from the contralateral ear was reconstructed if its counterpart was damaged or missing. Reconstruction required choosing a greyscale threshold, such that pixels taking a higher value were regarded as ‘bone’ and those taking a lower value were regarded as ‘non‐bone’ (i.e. air or soft tissue). This threshold was initially chosen by eye having compared a number of scans, but once selected, the same value was used for all prepared skull reconstructions. The mongrel and Chihuahua specimens included soft tissue and required different reconstruction thresholds. The proportion of images in the stack in which the object in question was outlined, and the density of points forming each outline, were varied according to the size and complexity of the structure. The resulting outlines were then manually corrected where it was clear that Stradview had failed to identify a boundary accurately. It was found that parts of the stapes, the smallest and thinnest of the structures reconstructed, were often not well resolved and required a lot of manual correction, especially of the crura.

**FIGURE 2:**
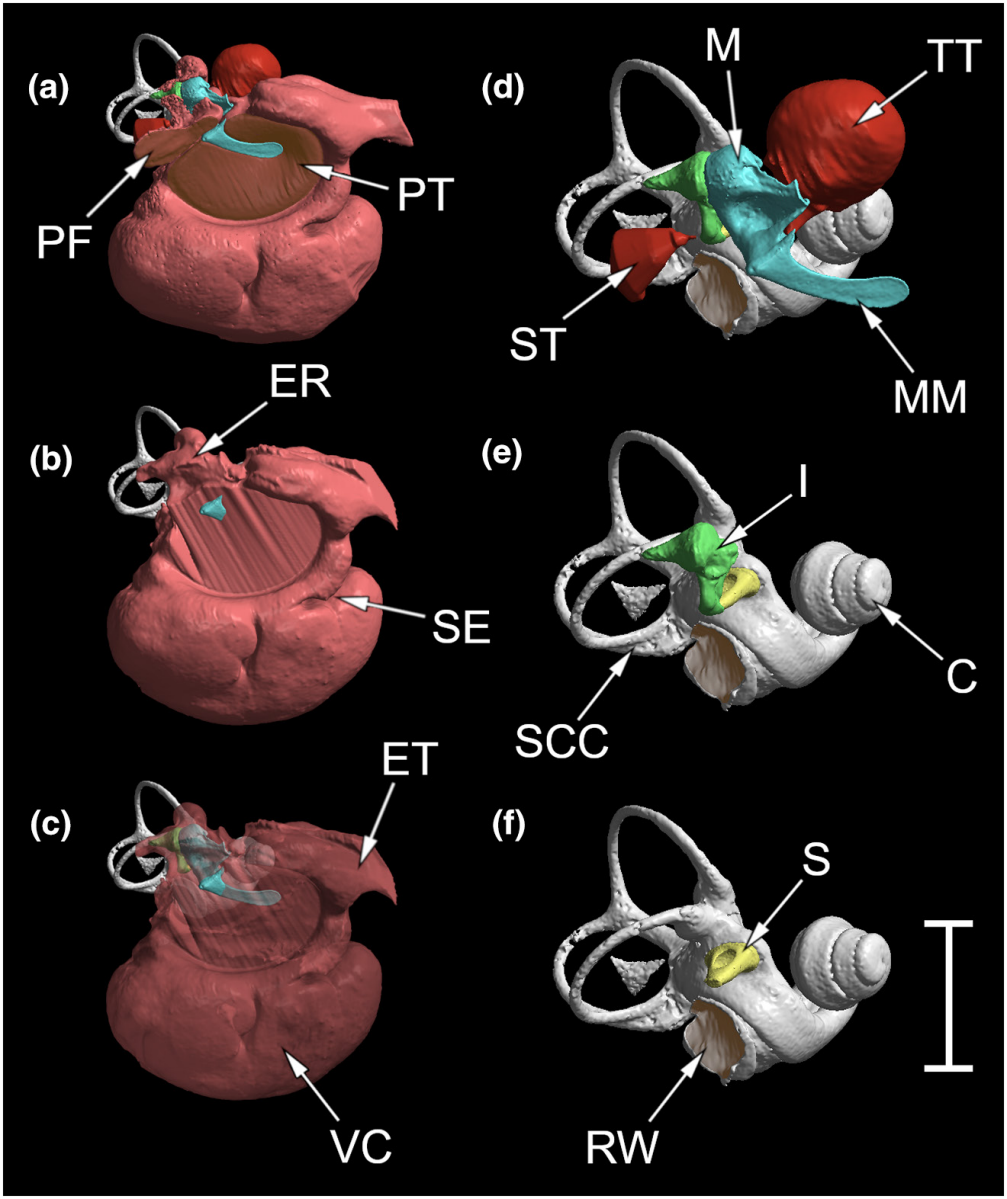
Reconstructions of the right middle ear and bony labyrinth of the mongrel, each from an approximately rostrolateral view. Reconstruction (a) shows soft‐tissue boundaries of the middle ear cavity (red); reconstructions (b) and (c) use bony landmarks. The septum (SE), visible as an invagination of the rostral middle ear cavity boundary, indicates the partial division between tympanic cavity (above) and ventral compartment (below), Reconstructions (d), (e) and (f) are magnified ×2 relative to a, b and c. The stapedius muscle belly was hard to distinguish from surrounding soft‐tissue structures, and its reconstruction is an estimate. C, cochlea; ER, epitympanic recess; ET, Eustachian tube; I, incus; M, malleus; MM, manubrium of malleus; PF, *pars flaccida* of tympanic membrane; PT, *pars tensa* of tympanic membrane; RW, round window; S, stapes; SCC, secondary *crus commune*; SE, partial septum; ST, stapedius muscle; TT, tensor tympani muscle; VC, ventral compartment of middle ear cavity. Scale bar 10 mm for reconstructions a, b, c and 5 mm for d, e, f.

Because 17 of our 19 specimens were dried skulls, soft‐tissue boundaries of their middle ear cavities had to be estimated from bony landmarks. The tympanic membrane was regarded as a flat surface contained within the bony tympanic annulus. The extension of the middle ear cavity into the Eustachian tube was abbreviated at an equivalent point in each scan (Figure 2c), which was where it emerges from the confines of the basicranium and, as visible in the mongrel scan, its lumen narrows dramatically. It was ensured through comparison that the estimated position of the round window was also consistent. Elsewhere, the boundaries of the middle ear cavity were taken to be the surrounding bone; no account was taken of the mucosal lining of the cavity which would exist in vivo. The subcavities for the tensor tympani muscle belly and the facial nerve were not included in the middle ear cavity reconstructions. Volumes of the three ossicles, the middle ear cavity (excluding the volume occupied by ossicles) and the bony labyrinth were all taken from Stradview reconstructions made in this way. The same bony landmarks were used in reconstructing the Chihuahua ear. For the mongrel, reconstructions made using these bony landmarks were compared with reconstructions made using the true soft‐tissue boundaries, which were visible in this scan (see Section 3.2).

The stapes reconstruction was oriented such that the plane of the footplate was perpendicular to the line of view, and the area of the stapes footplate was then measured as a flat surface. The malleus length from the tip of manubrium to the centre of the malleo‐incudal articulation (Hemilä et al., 1995) was also measured from the scaled CT reconstructions. Cochlear turns were measured from the proximal end of the basilar membrane, as estimated from the bony spiral laminae to the apex of the cochlear canal, following the method of Ekdale (2010).

To estimate the areas of the tympanic membranes, the jpeg stacks were imported into MicroView 2.5.0 (Parallax Innovations Inc.). Scaled reconstructions made in that program were reoriented such that the plane of the tympanic annulus was perpendicular to the line of view. The tympanic annulus in dogs is incomplete in the region of the *pars flaccida*, and the boundaries of the membrane here could not be ascertained. To obtain an area estimate, the longest diameter *d*_1_ of the tympanic annulus was measured from the reconstruction. Then, the maximum perpendicular distance *d*_2_ from this diameter to the perimeter of the tympanic annulus (caudoventrally) was measured. Following Nummela (1995), the tympanic membrane was assumed to be flat and elliptical in shape, and its area was estimated as π.(*d*_1_/2).*d*_2_, where *d*_1_ represents the major axis and *d*_2_ a semi‐minor axis of the ellipse. Values of the tympanic membrane area estimated in this way were compared with measurements made from reconstructions of the mongrel ear (see Section 3.2).

Skull measurements from the Chihuahua were made from CT reconstructions. For the other specimens, skull measurements were made directly using callipers. Condylobasal length (CBL) was measured between the most rostral extent of the premaxilla and the posterior occipital condyles. Maximum skull widths extended between the zygomatic arches. Interaural distances were measured between the openings of each bony external auditory meatus. Because of the irregular shape and inclination of the bony meatus in dogs, some subjectivity was involved in determining the points to measure between. Ossicles were dissected out of the mongrel ear, air‐dried and weighed on a Cahn C‐31 microbalance.

Skull and ear measurements for all specimens are available in the Supplementary Material. In producing the figures, some reconstructions were laterally inverted to make all appear to be of right ears.

### 2.3 Statistical methods

The main part of the statistical analysis was performed using data from 17 dog breeds, excluding the mongrel and the wolf. First, base‐10 logarithms were calculated for skull and ear measurement and Pearson correlation coefficients were computed. Following the arguments of Smith (2009), ordinary least‐squares linear regressions were chosen to model the scaling relationships between ear measurements and CBL, also calculated using logged data. The Lhasa Apso malleus lacked the tip of the manubrium, so its malleus volume was not included in the data set for statistical analysis.

The traditional phylogenetic tree structure is greatly complicated in dogs by multiple hybridization events during recent breed development (Parker et al., 2017). As noted by Schweizer et al. (2017), this makes it very difficult to apply phylogenetic correction to statistical analyses of canine morphology. Because of the very high *R* ^2^ values of most of the regressions examined here, the positions of the regression lines will in any case be relatively resistant to distortion through relatedness effects. For these reasons, phylogenetic correction was not attempted.

All statistical tests were performed in Microsoft Excel, using methods taken from Snedecor and Cochran (1967) and Sokal and Rohlf (1995).

## 3 RESULTS

### 3.1 Anatomical description of the mongrel middle ear

This initial description of the canine ear is based on the CT scan of the mongrel, an animal of intermediate size in which soft‐tissue structures could be visualized. Comparative comments will follow (Section 3.3).

**FIGURE 3:**
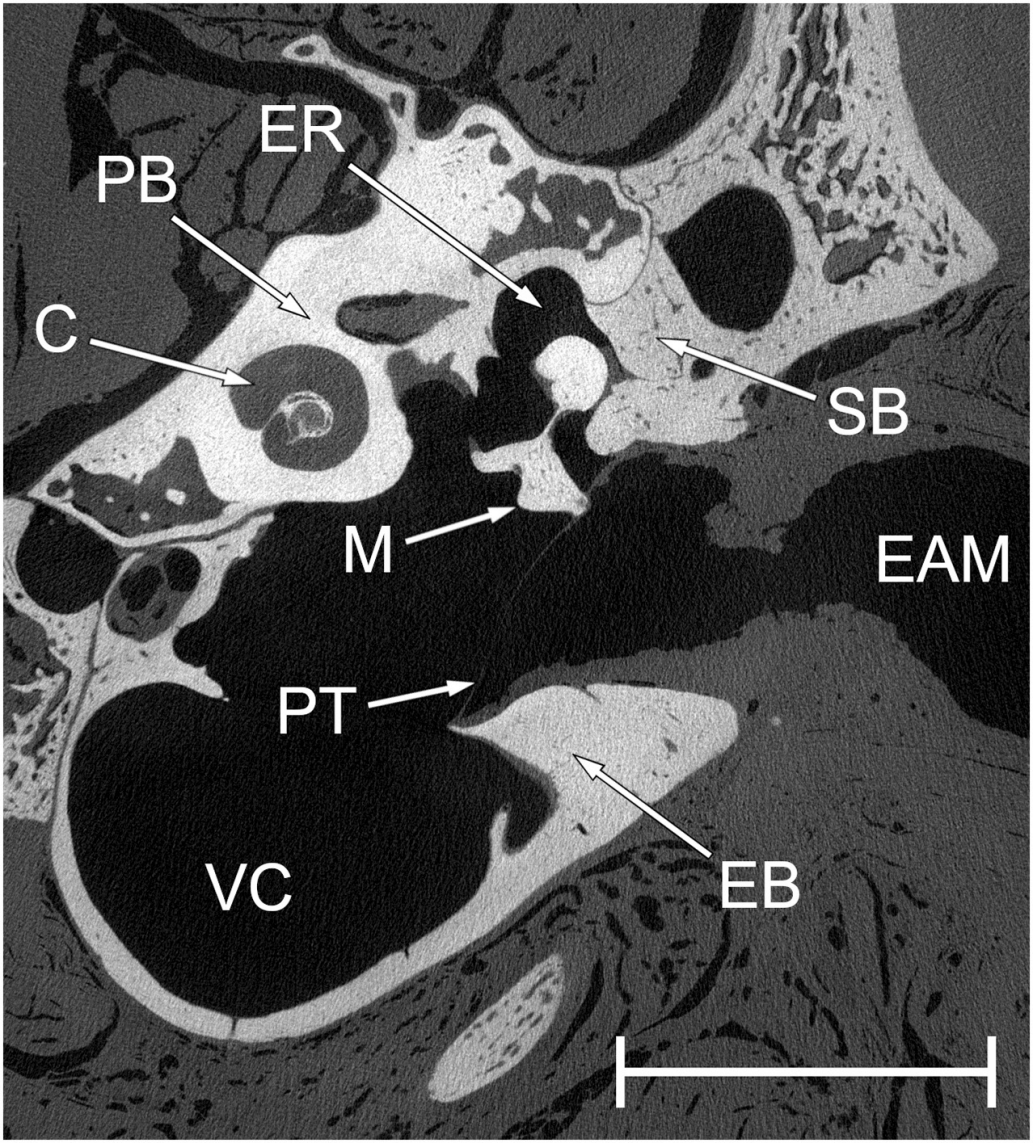
Tomogram cross‐section through the middle ear of the mongrel, to show the bones enclosing the cavity. Dorsal is upwards and lateral is to the right. The malleus section shows the head (dorsally, within the epitympanic recess), the lateral process (ventrolaterally, inserting into the tympanic membrane) and the muscular process (medially). C, cochlea; EAM, external auditory meatus; EB, ectotympanic bone; ER, epitympanic recess; M, malleus; PB, petrosal bone; PT, *pars tensa* of the tympanic membrane; SB, squamosal bone; VC, ventral compartment of middle ear cavity. Scale bar 10 mm.

Rostral to the posterior boundary of the bony external auditory meatus, sutures between temporal bones are visible (Figure 3). Here, the bulla is formed largely by the ectotympanic, which forms the bony annulus containing the tympanic membrane. Although other bony elements are known to contribute to the carnivore bulla (Wible & Spaulding, 2012), these could not be distinguished in the skulls examined. Dorsally, the middle ear cavity is covered by the particularly radiodense petrosal, which houses the bony labyrinth and the cavity for the body of the tensor tympani muscle. The squamosal contributes to the lateral wall of the epitympanic recess, and there is a very small contribution from the sphenoid to the roof of the tympanic cavity in the most rostral region, including over the origin of the Eustachian tube. Posterior to the external auditory meatus, the bones contributing to the walls of the middle ear cavity become indistinguishably fused.

The middle ear cavity (Figures 2a–c, 3) can be roughly divided into two halves. The ventral half is an empty, ovoid cavity which fills most of the externally visible auditory bulla. It is partially separated from the dorsal half by a septum rostrally and dorsomedially, but the two cavity components are elsewhere in wide communication. The dorsal half of the middle ear cavity, representing the tympanic cavity, is bounded laterally by the *pars tensa* of the tympanic membrane, and it contains the ossicles. It is irregular in shape, with projections rostrally (becoming the Eustachian tube), dorsally (a small epitympanic recess, containing the heads of the malleus and incus) and posteriorly (a tongue‐like extension covered laterally by the *pars flaccida* of the tympanic membrane). Rostral to the epitympanic recess, there is an almost spherical bony chamber within the petrosal which houses the tensor tympani muscle belly. This chamber is just dorsolateral to the cochlear spiral, the two being divided by a very thin layer of bone.

The carotid canal containing the internal carotid artery runs through the bone just medial to the tympanic cavity, but the canal and cavity do not communicate. There is no stapedial artery. The canal for the facial nerve is open to the tympanic cavity, just posterior to the long process of the incus.

The perimeter of the *pars tensa* of the tympanic membrane (Figures 2a, 3) follows an oval outline except for dorsocaudally, where it is not enclosed within the bony tympanic annulus. Its boundary here with the *pars flaccida* is almost straight and includes the lateral process of the malleus. The maximum inflection of the *pars tensa* at the tip of the manubrium was around 1.25 mm. The manubrium of the malleus is not symmetrically positioned within the *pars tensa* but is instead dorsal to its longest axis; its tip is rostral and dorsal to the centre of the membrane. Calculated as a flat surface in the plane of the tympanic annulus (see Section 2.2), the mongrel's *pars tensa* had an area of 59.8 mm^2^. Its true area, as a curved surface, was 62.2 mm^2^. The *pars flaccida* had an area of 11.3 mm^2^ when similarly treated as a flat surface, while its true curved‐surface area was 14.0 mm^2^. Of course, the shape of these membranes in vivo might well differ from what was found in this preserved specimen.

The malleus (Figures 2d, 3) has a small head. Its transversal lamina extends rostrally into a short, wide anterior process which is indistinguishably synostosed with the ectotympanic. The muscular process for the insertion of the tensor tympani muscle is conical, bent dorsally at the tip and very large. It is perforated near its base, ventrally, by a very narrow canal presumed to be for the chorda tympani nerve. The manubrium appears very substantial but the bone is internally hollow, its central cavity filled with some kind of soft tissue unidentifiable from the scans. It gently curves rostrally and has a flattened inserting margin, wider towards the tip. From a posterior view, the manubrium is triangular, one apex being the pronounced lateral process. There was a thin, spinous projection from the lateral process in the anterior direction in the mongrel specimen, occupying part of the boundary between *pars tensa* and *pars flaccida* of the tympanic membrane. The articulation facet for the incus is complex in shape, featuring two flattened facets dorsomedially and a convex facet caudoventrally.

The incus (Figure 2e) is robust, with processes of roughly the same length. The short process is contained within a posterior extension of the epitympanic recess, attached there by means of soft tissue. The long process supports a small, flat, oval lenticular apophysis for articulation with the stapes, upon a thin pedicle. The complex articulation facet for the malleus is the counterpart to the malleus facet described above. Dissection of the mongrel revealed that the ossicles were not fused to each other, the joints between them presumably being synovial.

The stapes (Figure 2f) is roughly triangular in dorsal view. It has a small head resembling a flattened cap. Near the head on the posterior crus is a slightly raised region for the insertion of the stapedius muscle. The oval footplate has a pronounced rim, deeper anteriorly and posteriorly. The footplate fits very snugly into the oval window, its central part bulging into the vestibule. The crura, footplate and neck are all deeply excavated and hence U‐shaped in cross‐section: the bone is very thin.

The malleus of the mongrel weighed 20.72 mg and the incus 9.34 mg. Based on the volumes obtained from the CT reconstructions, malleus density was 2.04 mg mm^−3^ and incus density was 2.09 mg mm^−3^. Unfortunately, the very delicate stapes was not retrieved intact.

The tensor tympani muscle (Figure 2d) is very large. Its approximately spherical muscle belly is contained within a bony recess in the petrosal. Its short tendon projects almost vertically downwards to insert into the muscular process of the malleus. The volume of the muscle was 55 mm^3^. The stapedius muscle originates within the facial canal but could not be reliably distinguished from the facial nerve in this specimen. A very small bony nodule was identified within the stapedius muscle which might have represented a skeletal element of Paaw.

It is beyond the scope of this paper to describe the inner ear in detail. From the reconstruction of the bony labyrinth (Figure 2e,f), the round window was found to be bean‐shaped and deeply inflected, and the cochlear spiral had 3.3 turns. A secondary *crus commune* was observed between the posterior and lateral semicircular canals.

### 3.2 Accuracy of measurements using bony landmarks

Volumes of the bony labyrinth and middle ear cavity had to be estimated using bony landmarks in all skull specimens. In order to assess accuracy, reconstructions were made of the ear of the preserved mongrel dog, based both on the same bony landmarks (Figure 2b) and on the soft‐tissue boundaries which could be clearly seen in the mongrel scan (Figure 2a). The most obvious differences in the reconstructions based on bony landmarks were that (1) the *pars tensa* of the tympanic membrane was taken to be approximately flat instead an inflected, curved surface; (2) the posterior extension of the middle ear cavity covered by the *pars flaccida* could not be identified; and (3) the Eustachian tube region was wider. The head of the malleus and the head and short process of the incus were partially exposed in the reconstruction using soft‐tissue boundaries (Figure 2a) due to the presence of either fluid or soft tissue between the ossicles and the roof of the epitympanic recess, with no intervening air‐space.

In the mongrel specimen, the reconstruction of the bony labyrinth using bony landmarks was just under 5% larger, and the reconstruction of the middle ear cavity was just under 7% larger, than the more accurate values measured using soft‐tissue boundaries. The tympanic membrane area estimated as an ellipse based on the dimensions of the bony tympanic annulus was 68.3 mm^2^, which is 10% larger than the true *pars tensa* area calculated as a curved surface.

### 3.3 Comparative description

**FIGURE 4:**
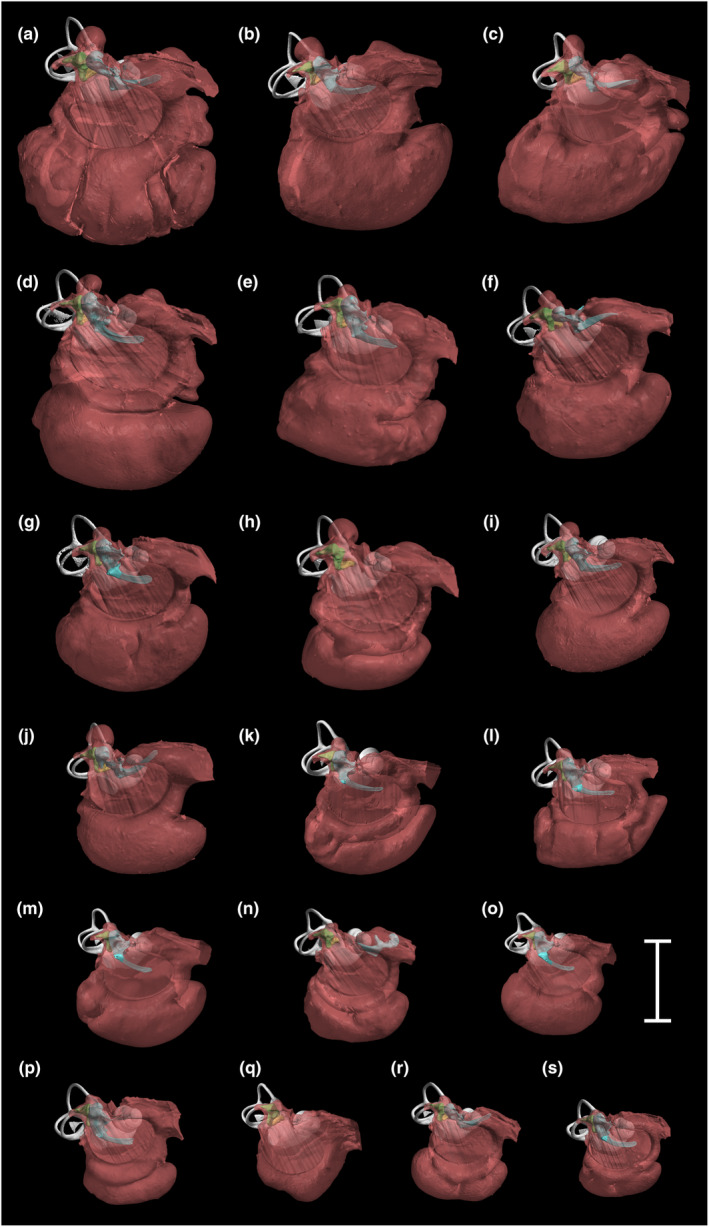
Reconstructions of the right middle ears and bony labyrinths of canids, each from an approximately rostrolateral view, to scale. The boundaries of the middle ear cavities (red) were determined using bony landmarks. Note that the malleus (blue) was displaced or missing in some of these ears. Other features can be identified with reference to Figure 2. a: St Bernard; b: Rottweiler; c: Saluki; d: wolf; e: Bloodhound; f: Pointer; g: mongrel; h: Alsatian; i: Boxer; j: English Bull Terrier; k: Dachshund; l: Poodle; m: Jack Russell Terrier; n: Lhasa Apso; o: Pekingese; p: Cavalier King Charles Spaniel; q: Boston Terrier; r: Pug; s: Chihuahua. Scale bar 10 mm.

The middle and inner ear structures of the canids (i.e. dogs plus wolf) varied considerably in size (see later), but the tympanic annuli, ossicles and bony labyrinths were morphologically very similar. The middle ear cavities were much more variable, particularly in the shape and relative size of the ventral compartment (Figure 4). Only the Boston Terrier had a simple cavity structure without a distinct ventral compartment (Figure 4q). The bone making up the ventral bullar wall in this breed was very thick, but spongy.

**FIGURE 5:**
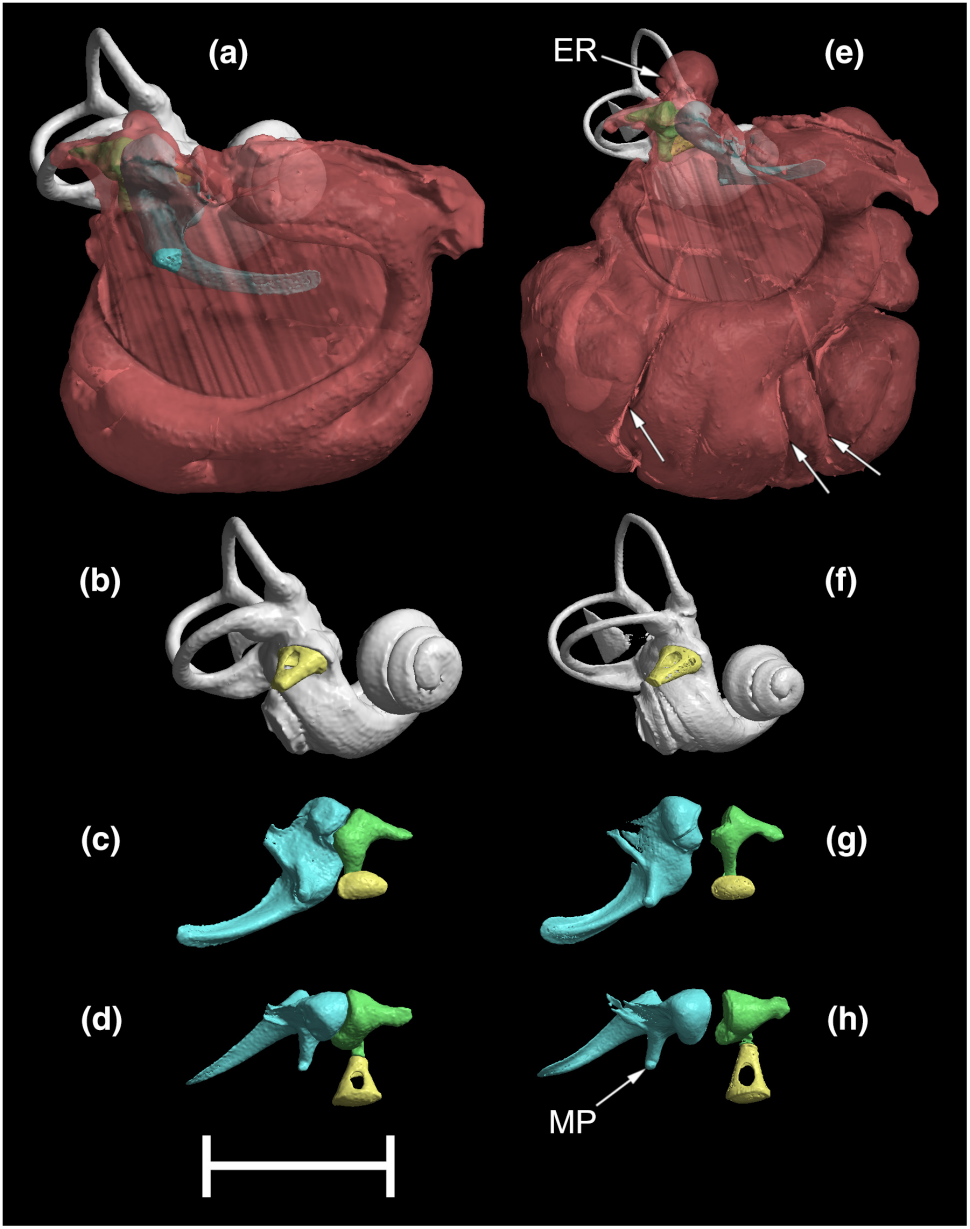
Reconstructions of the right middle ear and bony labyrinth of a Chihuahua (a–d) and a St Bernard (e–h). The 5 mm scale bar applies only to the Chihuahua reconstructions; the St Bernard reconstructions are not to scale but are represented at equivalent size for comparison. The malleus in the St Bernard specimen was dislodged slightly from its in vivo position and was disarticulated from the incus. (a), e: Reconstructions including the boundaries of the middle ear cavity, determined using bony landmarks and shown in translucent red, seen from approximately rostrolateral views; (b), f: bony labyrinth and stapes from approximately rostrolateral views; (c), g: malleus, incus and stapes from dorsomedial views; (d), h: malleus, incus and stapes from approximately dorsal views. The arrows in (e) show some partial septa in the ventral compartment of the middle ear cavity of the St Bernard. ER, epitympanic recess; MP, muscular process of the malleus. Other features can be identified with reference to Figure 2.

Although the incus tended to remain in its natural position, the malleus was often displaced in the dried skulls (Figures 4, 5), and sometimes the stapes was too. The articulations between ossicles and skull appeared identical in nature in all dogs where the ossicles remained in situ. All specimens had the characteristically curved and internally hollow manubrium. The tunnel through the base of the malleus muscular process was visible only in four of the dog breeds, plus the wolf. The spinous projection from the lateral process of the malleus was present in the wolf, but poorly developed or absent in the other specimens. The bony nodule within the stapedius muscle was not present in the Chihuahua in which soft tissue remained. Even if originally present, it would have been lost from the prepared skulls.

Considering the bony labyrinth, the English Bull Terrier had 2.9 cochlear turns, the Poodle 3.4 and the other canids fell somewhere in between. The anterior semicircular canal always had the largest radius of curvature, but the relative sizes of the canals differed. The Boxer and Pug had posterior canals with relatively small radii of curvature, in comparison with the anterior. All canids had a secondary *crus commune* between posterior and lateral canals.

The Chihuahua and St Bernard represented the two extremes of ear size among the dogs examined. Compared to the Chihuahua, the St Bernard's middle ear cavity volume was 14 times as large, its malleus and incus volumes were each around four times as large, its bony labyrinth volume was around three times as large, and its stapes volume, stapes footplate area and tympanic membrane area were all around twice as large. This shows that there are changes in relative proportions between the two breeds. When the reconstructions of the Chihuahua and St Bernard ears are compared side‐by‐side (Figure 5), the middle ear cavity of St Bernard can be seen to have a relatively expanded ventral compartment which is reinforced by several partial septa, running in an approximately vertical orientation. The St Bernard also has a relatively expanded epitympanic recess, dorsally, and its bony labyrinth includes relatively longer and narrower semicircular canals. The malleus and incus are remarkably similar in the two breeds, but the stapes is more elongated in the St Bernard (Figure 5h).

Using the ossicular density values obtained from the mongrel and the volumes from CT reconstructions, and excluding the Lhasa Apso which lacked an intact malleus, the malleus mass for the other breeds was calculated to range from 7.7 to 29.4 mg (mean 15.1 mg, SD 6.1 mg) and the incus mass from 3.5 to 15.5 mg (mean 7.7 mg, SD 3.3 mg). The largest malleus and incus were both from the St Bernard, the smallest malleus was from the Chihuahua and the smallest incus was from the Pekingese. The estimated malleus mass of the wolf was 27.0 mg, and its incus mass was 13.6 mg.

### 3.4 Statistical analyses

Correlation coefficients were used to establish whether the ear measurements were more closely correlated with condylobasal length (CBL), maximum skull width (MSW) or interaural width (IW), using logged data. Relationships with cephalic index, defined as 100 × (MSW/CBL), were also established, using logged data for the ear measurements only. All but one of the ear measurements correlated more closely with CBL than with either of the skull widths or the cephalic index (Table 1). The sole exception was stapes volume, which was more closely correlated with maximum skull width than with the other skull measurements. Correlation coefficients relating to stapes volume measurements were lower than for any other ear measurement, probably reflecting difficulties in measuring this tiny ossicle accurately (see Section 2.2).

**TABLE 1:** Pearson correlation coefficients (*r*) between anatomical variables, for 17 dog breeds. All of these correlations were calculated using the base‐10 logarithms of the variables shown, except for the dimensionless cephalic index.

	Maximum skull width, mm	Interaural width, mm	Middle ear cavity volume, mm^3^	Malleus volume, mm^3^	Incus volume, mm^3^	Stapes volume, mm^3^	Bony labyrinth volume, mm^3^	Tympanic membrane area, mm^2^	Stapes footplate area, mm^2^
Condylobasal length, mm	0.731	0.828	0.938	0.927	0.892	0.560	0.903	0.964	0.881
Maximum skull width, mm	‐	0.940	0.784	0.787	0.764	0.619	0.801	0.745	0.788
Interaural width, mm	‐	‐	0.852	0.828	0.799	0.591	0.827	0.837	0.808
Cephalic index	‐	‐	−0.727	−0.699	−0.686	−0.311	−0.666	−0.783	−0.634

**FIGURE 6:**
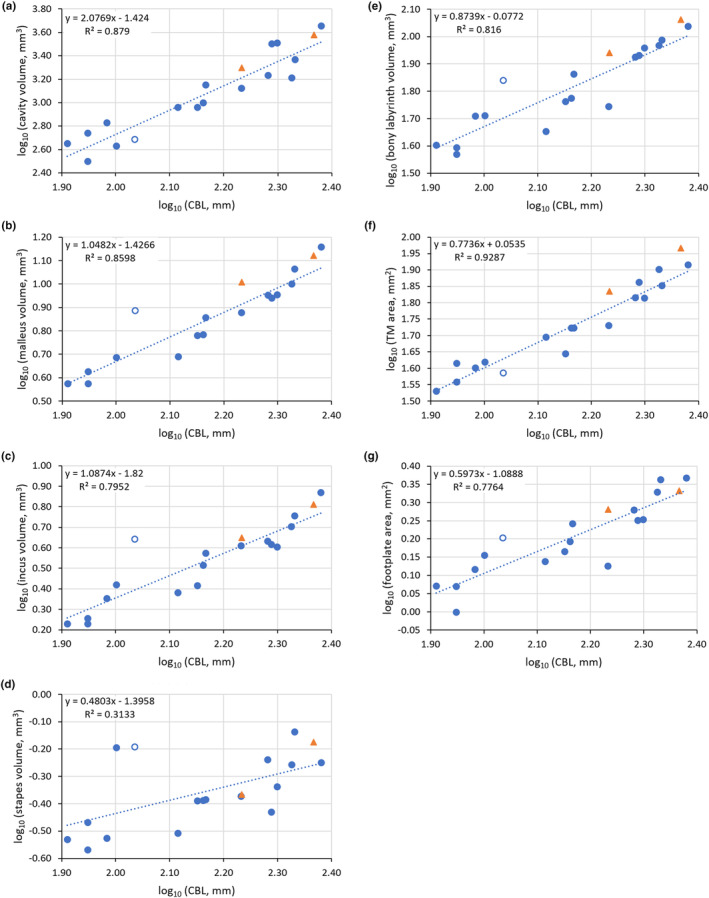
Relationships between the base‐10 logarithms of middle and inner ear measurements and condylobasal length (CBL). (a) Middle ear cavity volume; (b) malleus volume; (c) incus volume; (d) stapes volume; (e) bony labyrinth volume; (f) tympanic membrane (TM) area; (g) stapes footplate area. The blue circles represent the 17 dog breeds. Least‐squares regression lines using these data are shown, and the corresponding equations are given. The Cavalier King Charles Spaniel (open circles) has relatively large ossicles. The orange triangles represent the mongrel (smaller CBL) and wolf (larger CBL): these are included for comparison and were not used to calculate the regression relationships.

The regressions of the various ear measurements on CBL, using logged data, are shown in Figure 6 and Table 2. In every case, the slope of the regression line is positive, but significantly lower than the value expected from an assumption of isometry (all *p* < 0.001). This indicates that larger dogs tend to have absolutely larger, but relatively smaller, ear structures than smaller dogs, i.e. ear structures scale with negative allometry. The slope coefficient for the regression of cavity volume on CBL, around 2, is much higher than that for malleus, incus and bony labyrinth volumes, which are all around 1. Larger dogs therefore have relatively large ear cavities, compared to these other ear components.

**TABLE 2:** Results of least‐squares regression analysis, performed on base‐10 logarithms of the variables shown and using log_10_ (condylobasal length, mm) as the *x* axis value, for 17 dog breeds. The expected slope is based on an assumption of isometry; the probability that the population slope matches the expected value is <0.001 in all cases.

	Middle ear cavity volume, mm^3^	Malleus volume, mm^3^	Incus volume, mm^3^	Stapes volume, mm^3^	Bony labyrinth volume, mm^3^	Tympanic membrane area, mm^2^	Stapes footplate area, mm^2^
Expected slope	3	3	3	3	3	2	2
Measured slope	2.077	1.048	1.087	0.480	0.874	0.774	0.597
Intercept	−1.424	−1.427	−1.820	−1.396	−0.077	0.054	−1.089
R^2^	0.879	0.860	0.795	0.313	0.816	0.929	0.776

Standardized residuals (individual *y* axis residuals divided by the standard deviation of all *y* axis residuals) were calculated for each regression relationship with CBL, to identify outliers. Values above 2, indicating substantial deviations from the expected relationships, were found for the Cavalier King Charles Spaniel, which had relatively large ossicular and bony labyrinth volumes (malleus 2.77, incus 2.92, stapes 2.05, bony labyrinth 2.15). The Boston Terrier had a relatively large stapes volume (2.18), the Poodle had a relatively small tympanic membrane area (−2.18) and the English Bull Terrier had a relatively small stapes footplate area (−2.38).

Although measurements from the mongrel and from the wolf were not used in calculating correlation coefficients or regression relationships, points representing these specimens are shown in each panel of Figure 6. These points all fall relatively close to the regression lines; only the tympanic membrane area of the wolf had a standardized residual over 2 (2.47). This contributed to the wolf having the highest tympanic membrane to stapes footplate area ratio of any animal studied, taking a value of 43.2. The Cavalier King Charles Spaniel had the lowest area ratio (24.2); the mean value for all 19 specimens was 34.1.

## 4 DISCUSSION

Qualitatively, our reconstructions of the ear structures of the mongrel are consistent with descriptions of the generalized dog ear in the literature (Cole, 2010; Ekdale, 2013; Evans, 1993; Getty et al., 1956). Although falling into Fleischer's (1978) ‘transitional’ category in overall morphology, the canid malleus is characterized by a thick, curved (‘banana‐shaped’) manubrium and very prominent muscular process, as previously described in the red fox *Vulpes vulpes* (Malkemper et al., 2020). The synostosis between the anterior process of the malleus and the bony wall of the tympanic cavity, found here in all specimens in which the malleus was still in situ, was noted as a carnivore feature by Wible and Spaulding (2012). A small piece of cartilage or bone within the tendon of the stapedius muscle, known as the skeletal element of Paaw, has been reported in many mammalian species but apparently never in canids (Wible et al., 2021). It remains to be determined whether the bony nodule found within the stapedius muscle belly of the mongrel only is a feature of the normal dog ear.

Quantitatively, middle ear cavity volumes of 18 mesaticephalic dogs, measured from much lower‐resolution CT scans by Defalque et al. (2005), ranged between around 0.2 and 2.7 mL and the six measurements made by Kirikae (1960) fell within that range. Our volumes ranged from 0.32 mL in the Chihuahua to 4.54 mL in the St Bernard, apparently the highest volume yet measured in a dog. The *pars tensa* area and ossicular masses reported by Nummela (1995) were similar to the values we obtained in our mongrel specimen, which was a medium‐sized dog. The oval window area reported by Hemilä et al. (1995), from the same specimen examined by Nummela, was very similar to our mongrel's stapes footplate area.

The *pars tensa* areas of the Chihuahua and St Bernard estimated from tympanic annulus dimensions here were 21% and 49% larger, respectively, than the tympanic membrane areas measured as flat surfaces from dissected specimens by Heffner (1983). The measurement method used here was found to produce an overestimate of the true *pars tensa* area in the mongrel (see Section 3.2), but differences between individuals are in any case to be expected.

Fleischer (1973) noted that mean eardrum areas, stapes footplate areas, ossicle sizes and bullae were all smaller in six domestic dogs (two Boxers, two Borzois, two Standard Poodles) than in six wolves, which he attributed to the effects of domestication. Although our single wolf specimen did tend to have slightly larger ear structures than expected, our findings suggest that differences in skull size will have contributed significantly to the smaller ears Fleischer found in his dogs. Fleischer also noted a higher tympanic membrane to stapes footplate area ratio in wolves than dogs; among our specimens, the wolf did indeed have the highest ratio. Analogous findings were reported by Fleischer in *Lama* species, and by Burda (1985) in a comparison of wild and laboratory rats. Although one might infer that auditory acuity is reduced with domestication, the link between the area ratio and acuity is questionable (Mason, 2016: see Section 4.2).

Schweizer et al. (2017) found between 3 and 3.5 cochlear turns among their sample of domestic dogs, wolves and dingoes, measuring to the nearest quarter‐turn. This is consistent with the findings of the present study. Schweizer et al. found the lowest number of turns (3) in the Dachshund and Labrador Retriever among modern breeds, but they did not examine a Bull Terrier; our Dachshund had 3.2 turns. Although we found the relatively small size of the posterior semicircular canal compared to the anterior in the Boxer and Pug to be visually striking, Schweizer et al., using a geometric morphometric approach, found the lateral semicircular canal to be more variable than the other two among the canids they studied.

### 4.1 Scaling of ear structures

Among terrestrial mammals in general, there is an inverse correlation between interaural widths and the limits of high‐frequency hearing, attributed to the requirements for sound localization (Heffner et al., 2001; Heffner & Heffner, 2003). Given that smaller middle ear structures should favour the transmission of higher‐frequency sound to the cochlea, we would expect smaller mammals to have smaller middle ear structures. This is indeed the case in absolute terms although across mammals in general, the relationship between the size of middle ear structures and various indices of body size is negatively allometric (Mason, 2001, 2013; Mennecart et al., 2020; Nummela, 1995; Nummela & Sánchez‐Villagra, 2006). Accordingly, we found that dog ear structures have a negatively allometric relationship with condylobasal length (CBL), a measure of skull length (Table 2). In other words, smaller dogs have absolutely smaller, but relatively larger, ear structures than larger dogs.

Perhaps surprisingly, almost all ear measurements correlated more strongly with CBL than with either of our skull width measurements (Table 1). Wayne (1986) found that basicranial length correlates very closely (Pearson *r* = 0.97) with total skull length in domestic dogs, scaling with slight negative allometry. The bullae, on the basicranium, are therefore likely to get relatively smaller as dog skulls get longer, consistent with our finding that middle ear cavity volume scales with CBL with negative allometry. Structures within the middle ear scale with more pronounced negative allometry, but still show a close relationship with skull length.

Although large dogs have skulls of similar proportions to wolves, small dogs tend to have relatively wider skulls. For dogs of a given maximum skull length, skull widths are much more variable than basicranial lengths (Wayne, 1986). Overall, the tighter correlation of ear dimensions with skull length rather than with either of our measures of skull width may relate to how skull structure as a whole scales in dogs of different breeds. As might be expected, correlation coefficients between ear measurements and interaural widths were generally higher than with maximum skull (zygomatic) widths (Table 1). We measured interaural widths between the openings of the bony parts of the external ear canals, but a tighter correlation might have been achieved if we had been able to measure between pinna openings.

Guérineau et al. (2022) cited research suggesting that variability in sound localisation acuity among mammals is linked to the field of binocular vision (Heffner & Heffner, 1992), and went on to note that the visual field, in dogs, may be constrained by skull shape (dolichocephaly versus brachycephaly). This implies that there may be selective pressures on aspects of canine audition, potentially including bony ear parameters, arising from differences in relative skull length, but there is as yet no solid data supporting this intriguing hypothesis.

From our regression analyses, the breed that stood out most was the Cavalier King Charles Spaniel, which had larger ossicular and labyrinth volumes than expected for its skull length. Its ossicles considered collectively were 60% larger in volume than expected, its labyrinth just over 35% larger. The ossicles did not, however, differ noticeably in morphology to those of the other dogs. Since only one specimen was investigated we cannot be certain that this is typical of the breed, but it is interesting to note that cases of primary secretory otitis media are disproportionately common in Cavalier King Charles Spaniels (Cole, 2012; Stern‐Bertholtz et al., 2003), suggesting that there may be something unusual about their middle ears. Cole et al. (2015) commented that these dogs appeared to have ‘small and shallow’ bullae, which could potentially be linked to the condition if drainage of fluids from the Eustachian tube is impeded. Relative to skull length, our specimen had a middle ear cavity volume that fell slightly below the regression line (Figure 6a), but nothing stands out regarding its cavity morphology (Figure 4p). Other authors have found a link between primary secretory otitis media and abnormal soft‐tissue morphology in the nasopharyngeal region (Hayes et al., 2010), which would not be visible in our scans.

### 4.2 Middle ear dimensions and canine audiograms

A mean audiogram for 11 dogs was presented by Lipman and Grassi (1942), looking at frequencies from 125 to 8 kHz only. Lipman & Grassi did not report the sizes or breeds of their subjects. Canine hearing ranges were later established by Heffner (1983), who used water as a reward to train dogs to respond to tones presented to them at different frequencies and intensities. Heffner thus obtained complete behavioural audiograms for a Chihuahua, a Poodle, a Dachshund and a St Bernard (Figure 1). The poodle weighed just under 7 kg (Henry Heffner, pers. comm., 2022), so it was of medium size for this very variable breed. Heffner also established the high‐frequency hearing limit of a Pointer. The dogs studied were all 1–4 years old.

**FIGURE 7:**
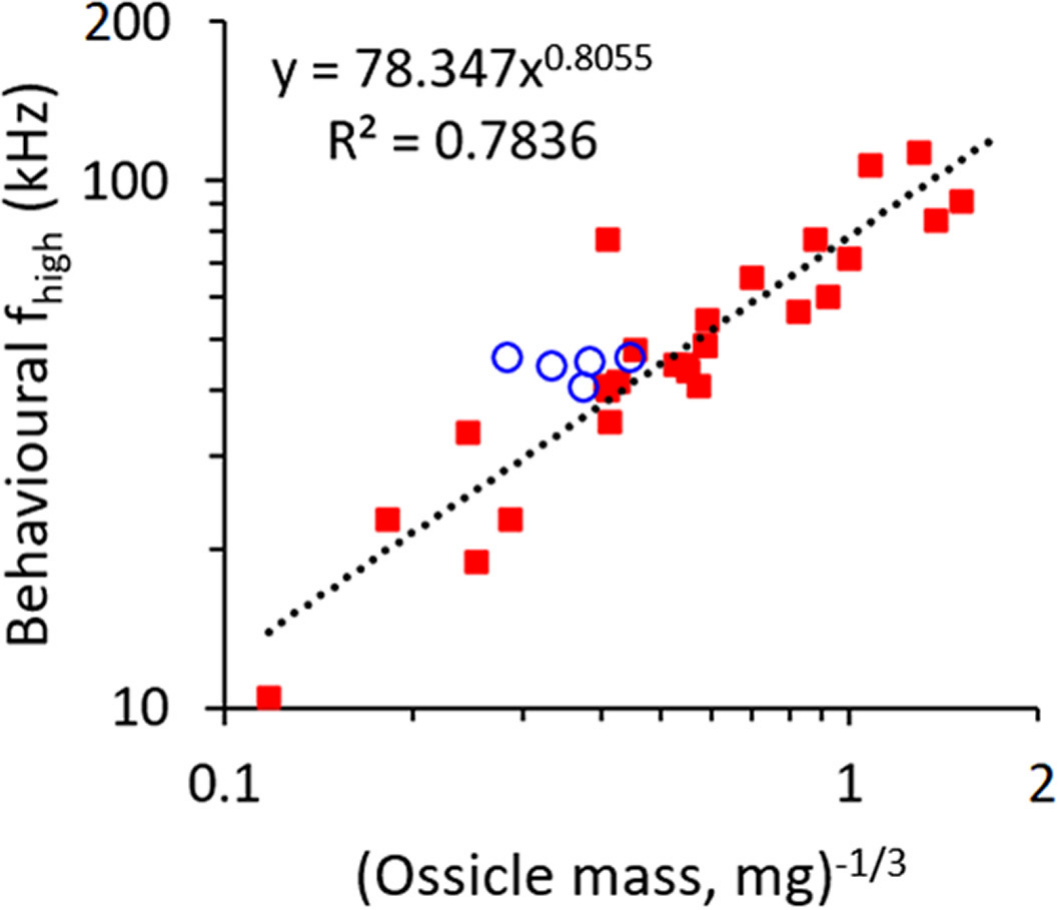
The high‐frequency limits of hearing at 60 dB SPL (f_high_), determined from behavioural audiogram data, plotted against the reciprocal of the cube root of malleus plus incus mass. Note that smaller ossicles are further to the right on the *x*‐axis. Data for mammals in general (red squares) were collected from direct measurements and from the literature by Hemilä et al. (1995), who created the original version of this graph. The single point representing a dog from that paper has been removed and replaced with points representing the five dog breeds tested by Heffner (1983); estimated ossicular masses for these dogs come from the present study. Dog breeds are represented as blue circles. The regression line was calculated for the Hemilä et al. data only, and its equation is shown.

Based on data from a wide variety of mammals from mice to elephants, Hemilä et al. (1995) found a strong relationship between ossicular mass and high‐frequency hearing limits. Even the difference in size between a Chihuahua and a St Bernard is small relative to the size‐range of mammals in general, and dogs overall fit the general mammalian relationship relatively well, with smaller dogs falling closer to the regression line than larger ones (Figure 7). Small dogs were also found to fit the relationship between functional interaural width and upper frequency of hearing better than the St Bernard, which seems to have slightly better high‐frequency hearing than expected (Heffner, 1983). However, despite substantial differences in body size, the upper and lower hearing limits in the Chihuahua and St Bernard were found to be very close (Figure 1 and Table 3). Can models of middle ear function based on considerations of acoustics help to explain this?

**TABLE 3:** Observed behavioural hearing limits at 60 dB SPL, taken from Heffner (1983), together with predictions made by the model of Hemilä et al. (1995) using anatomical data from the present study.

Breed	CBL, mm	Observed low‐frequency hearing limit, Hz	Observed high‐frequency hearing limit, kHz	Predicted high‐frequency hearing limit, kHz
Chihuahua	88.7	95	47	50.2
Poodle	141.6	<60	46	45.3
Dachshund	145.2	60	41	41.7
Pointer	191.2	‐	45	36.6
St Bernard	240.0	90	47	30.9

*Note*: Low‐frequency hearing limits are approximations based on Heffner's audiograms. Dog breeds are listed in ascending order by condylobasal length (CBL), as measured in the present study.

Low‐frequency sound transmission through the middle ear is dominated by acoustic compliance, which in a range of mammalian species has been found to be affected by the middle ear cavity volume and the stiffness of the tympanic membrane and ossicles (Ravicz et al., 1992; Ravicz & Rosowski, 1997). Following the analysis of Mason (2016), because middle ear cavity volume is 14 times larger in the St Bernard than in the Chihuahua, we would expect middle ear cavity compliance to be 14 times greater. If cavity compliance dominates overall compliance, the low‐frequency acoustic impedance of the middle ear will be nearly 23 dB lower. Volume velocity of a vibrating structure (i.e. volume displaced per unit time, in mm^3^ s^−1^) is equal to sound pressure divided by acoustic impedance. The *pars tensa* area is 2.3 times larger in the St Bernard, and so if we assume that it moves like a piston we can calculate that for any given sound pressure its velocity (in m s^−1^) at low frequencies should be 14/2.3 = 6 times greater than in the Chihuahua, equating to nearly 16 dB. The audiograms obtained by Heffner (1983) show that both St Bernard and Chihuahua have a 60 dB SPL low‐frequency hearing limit of around 90 Hz, but if the St Bernard enjoyed a 16 dB reduction in low‐frequency hearing thresholds, its low‐frequency limit should be more like 40 Hz.

This discrepancy could be explained if tympanic‐ossicular compliance, rather than cavity compliance, has the larger effect on overall middle ear impedance at low frequencies in dogs. This is known to be the case in domestic cats (Ravicz et al., 1992), and the dimensions of the cat middle ear (Huang et al., 1997) are close to that of the poodle. If tympanic‐ossicular compliance does indeed dominate low‐frequency impedance, at least in larger dogs, an expanded middle ear cavity volume would only have a very limited impact on hearing.

At mid‐frequencies the audiograms of the four breeds separate (Figure 1), but this does not seem to be linked to body size. The Poodle has the lowest thresholds for all frequencies below around 20 kHz. The anatomical area ratio between tympanic membrane and stapes footplate has, together with the ossicular lever ratio, been used to calculate the impedance transform ratio of the middle ear, which has in turn been linked to hearing sensitivity in mammals (Hunt & Korth, 1980; Rado et al., 1989; Webster & Webster, 1975). Contrary to what adherents of this view would predict, the area ratio of the Poodle, 30.1, is actually the lowest of any of the breeds for which we have audiograms. Mason (2016) presents a critical analysis of the area/lever ratio concept.

Turning to high‐frequency hearing, Hemilä et al. (1995) constructed an electrical analogue model of the middle ear of mammals based on just three anatomical parameters: tympanic membrane area, combined malleus + incus mass and a measure of malleus length which extends from manubrium tip to the centre of the malleo‐incudal articulation (see that paper for details of the model). These authors used their model to generate reasonably accurate predictions of behavioural high‐frequency hearing limits at 60 dB SPL across a wide range of mammals, including a prediction of 38.8 kHz in a dog of unspecified breed. Using data from the present study, the predictions from the same model are that the Chihuahua should have a substantially higher upper hearing limit than the St Bernard: 50.2 kHz as compared with 30.9 kHz (Table 3). This was not found to be the case by Heffner (1983).

In fact, the assumptions behind ‘lumped element’ models of middle ear function, such as the one created by Hemilä et al. (1995), break down at high frequencies where wavelengths become small relative to the dimensions of individual ear structures (Fletcher, 1992). At such frequencies, the tympanic membrane vibrates in complex modes, the ossicular chain can no longer be assumed to vibrate as a stiff, solid unit and the exact geometry of the middle ear cavity becomes important. As noted by Lavender et al. (2011), smaller ears inevitably result in predictions of higher‐frequency upper hearing limits within the Hemilä et al. model, so the good correspondence between model predictions and observed limits among mammals in general, across a wide range of body sizes, might be based on correlation as opposed to accurate modelling of vibratory behaviour. If the middle ear actually works as a transmission line (de La Rochefoucauld et al., 2010; Overstreet & Ruggero, 2002; Puria & Allen, 1998; Ravicz et al., 2008), there may be no clear link between gross anatomical structure and the upper limits of middle ear sound transmission.

Behavioural audiograms may be strongly influenced by factors other than middle ear sound transmission (Ruggero & Temchin, 2002), complicating any attempt to predict hearing from middle ear structure. The pinnae are very variable in different dog breeds, but Heffner (1983) tested his Dachshund with pinnae taped over the head or left to flop, and found only very small differences in thresholds (<3 dB) at high frequencies. Like the middle ear structures, the bony labyrinths of the dogs examined here were similar in morphology but very different in size, and there is no obvious reason to imagine that they would constrain hearing to essentially the same frequency range in all breeds. Perhaps the hearing ranges of dogs are tightly constrained by some other feature of their auditory system which did not change with the bony elements of the skull, under the artificial selection imposed by humans.

Before abandoning the attempt to relate ear dimensions to auditory function in dogs, we must consider the possibility that the behavioural audiograms collected by Heffner (1983) are not representative of those breeds as a whole. Heffner's results were obtained from just one dog per breed, but individual differences can be quite substantial in behavioural hearing studies. For example, when three red foxes were tested at 46 kHz, around the upper limit of hearing of the dogs examined by Heffner, Malkemper et al. (2015) recorded their thresholds at 21, 44 and 54 dB SPL. As Strain (2012) noted, a more comprehensive study is required before we can say for sure that there are no breed‐specific differences in hearing in dogs. Perhaps a reassessment will reveal that there are, in fact, differences in the hearing ranges of large and small breeds, as Galton (1883) had believed.

## 5 CONCLUSION

Domestic dogs vary considerably in skull size and shape, as a result of centuries of domestication and human selection. It is shown here that middle ear structures and bony labyrinths scale more closely with skull length than with skull width in dogs. Although relatively smaller, the ear structures of the biggest breeds are substantially larger in absolute terms than those of the smallest. Despite these differences in dimensions, middle and inner ear morphologies are similar across dogs of very different sizes, with ossicular morphology particularly well‐conserved.

Based on a comparison of cats, dogs and foxes, Malkemper et al. (2020) concluded that while the dimensions of ear structures do seem to tell us something about the hearing of mammals in crude terms, they cannot be used to predict more subtle audiogram differences between species of similar size. By looking at different dog breeds one can, in effect, control for more conflating variables than when making interspecific comparisons. Despite this, our finding is essentially the same—there is no clear relationship between ear size and behavioural hearing limits, even when comparing breeds as distinct as a Chihuahua and a St Bernard. However, before we can positively conclude that intraspecific differences in middle ear structure have no impact on hearing in dogs, canine audiograms should be re‐tested using more animals, to confirm that there really are no differences in hearing between dogs of such different sizes.

## AUTHOR CONTRIBUTIONS

MJM: conceptualization, methodology, investigation, formal analysis, writing—review and editing, supervision. MAL: investigation, formal analysis, writing—original draft.

## Supporting information


Data S1.

## Data Availability

The CT data that support the findings of this study are openly available in Zenodo at http://doi.org/10.5281/zenodo.10410546.
